# Service provider perspectives on implementing the NSW Get Healthy at Work program

**DOI:** 10.1080/17482631.2021.1945205

**Published:** 2021-07-05

**Authors:** Anne C. Grunseit, Erika Bohn-Goldbaum, Margaret Thomas, Rochelle Seabury, Chris Rissel, Melanie Crane

**Affiliations:** a The Australian Prevention Partnership Centre, Glebe, Australia; b Sydney School of Public Health, The University of Sydney, Camperdown, Australia; c NSW Ministry of Health, Glebe, Australia

**Keywords:** Partnerships, health promotion, lifestyle-related disease, workplace health program, process evaluation, qualitative

## Abstract

**Purpose**: One approach increasingly used by governments to deliver on public initiatives is to partner with private enterprise through public–private partnerships. This study is a qualitative process evaluation of an Australian state-wide workplace health programme “Get Healthy at Work” from the currently under-researched perspective of the private service providers. **Methods**: Semi-structured interviews were conducted with nine service providers. Interviews were transcribed and analysed inductively. **Results**: Service providers reported an alignment of motives and skills between the programme and their organizations as a benefit of the partnership. However, they also described misalignments: between the potential and realized value of the programme to businesses and service providers; the programme cycle and business operational processes; and the capacity building approach and businesses’ expectations of the service.**Conclusions**: Although several hallmarks of a well-functioning private–public partnership were evident, misalignments of process and expectations challenged sustained partnership involvement by providers. Careful consideration must be given to the ongoing management functioning of cross-sector engagement and partnering in health promotion practice in order to ensure public health goals are being met, but also that the model is mutually sustainable.

## Introduction

More than 70% of all deaths world-wide are related to chronic preventable diseases including diabetes, cardiovascular disease and cancer (World Health Organization, 2017). Conservative estimates suggest the cumulative global burden of non-communicable diseases (NCDs) would lead to a global loss of 47 USD trillion in the decades between 2015 and 2035 (Muka et al., 2015). Given many of the determinants of these diseases are ecological rather than individual, prevention often requires government intervention and health promotion implemented at scale (Marteau et al., 2012). The challenge many governments face, however, is how to best intervene for the optimal cost-benefit ratio with the shortest lead-in time. Health promotion initiatives which capitalize on existing services and programme delivery infrastructure may mobilize resources efficiently for rapid implementation at the general population level.

Workplaces are a growing area of interest as a setting for health promotion with potential to reach a high proportion of the adult population (The World Bank, 2017). There is evidence that multi-component workplace health promotion (WHP) programmes to promote physical activity, nutrition or both are effective at reducing overweight and obesity, increasing physical activity and improving healthy eating (Anderson et al., 2009). A large study across 20 organizations in the US showed that higher scores on an index summarizing workplace measures for heart health in terms of policies, programmes and “culture of health” were associated with lower rates of high blood pressure, high cholesterol and cardiovascular disease (Goetzel et al., 2007). A literature review of programmes integrating health promotion and traditional occupational health and safety similarly found significant improvements in blood pressure and cholesterol with implementation (Anger et al., 2015). Further, there is potential financial benefit to businesses providing effective WHP programmes (Astrella, 2017; Grossmeier et al., 2016). Previous research suggests successful WHP programmes are characterized by strong commitment from senior leaders, are tailored to worker needs and comprise a multi-faceted approach which addresses individuals, policy and the environment (Chau, 2009; Goetzel et al., 2007; Hector & St George, 2013; World Health Organization & Burton, 2010).

In Australia, whilst federal and state governments have been active in health promotion for the prevention of lifestyle-related chronic disease among the general population in schools and children’s services (Hardy et al., 2010; Welsby et al., 2014; Wiggers et al., 2013) through social marketing campaigns (King et al., 2013; Kite et al., 2018; O’Hara et al., 2016) and even the home (Blackford et al., 2016), they have not until recently programmatically targeted the workplace setting (Grunseit et al., 2016). Implementing WHP programmes at scale can be challenging given the considerable variation across sites in terms of business size, demographic profile, geographic location and internal culture and support for workplace health. Creating a sustainable, government-led WHP programme would therefore require considerable flexibility as well as a mechanism for implementation at scale which incorporates the aforementioned characteristics of success (Cahalin et al., 2015).

One approach increasingly used by governments to deliver on public initiatives is to partner with private enterprise (Batley & McLoughlin, 2010; Osborne, 2000). Partnering with private businesses may offer solutions that extend programme reach, offer expanded resource access, and generate employment opportunities. While all partnerships undoubtedly involve some kind of cooperation for mutual benefit, private–public partnerships (PPPs) are commonly characterized by the provision of government funding to the private sector, in order to provide a service or product. In health, they have most often been used for delivering large health infrastructure projects but are atypical for health promotion interventions (Johnston & Finegood, 2015). There are some examples, however, in chronic disease prevention such as the UK’s Public Health Responsibility Deal (Durand et al., 2015) and the Australian Food and Health Dialogue (Elliott et al., 2014). PPPs can potentially facilitate efficient and sustainable delivery of large-scale public health programmes, by harnessing the networks and implementation-ready resources of private enterprise providers (Schell et al., 2013).

### The GHaW PPP

In 2015, the New South Wales (NSW) government implemented a workplace health programme, Get Healthy at Work (GHaW) (Khanal et al., 2017), to encourage businesses to participate in prevention of lifestyle-related chronic disease among workers. The state-wide programme was funded through a federal government initiative (Grunseit et al., 2016). NSW Health entered into a partnership with WorkCover NSW (now SafeWork NSW https://www.safework.nsw.gov.au/) to jointly develop and implement GHaW. WorkCover NSW had existing state-wide engagement with NSW employers, access to NSW industry networks for occupational health and safety regulation and understanding of business operating environments. In order to implement the programme quickly at scale, Provider Organizations (POs) were contracted by WorkCover to implement certain aspects of the programme through a contractual arrangement with the NSW Government (Osborne, 2000). Communication channels operated between the government agencies (Ministry of Health and WorkCover programme managers) and POs, regarding modifications to the programme, administrative processes and feedback. A comprehensive, independent evaluation was designed to examine whether the objectives of the programme were being met, and explore the viability of the unique intersectoral arrangement which delivered it, including from the persepective of private enterprise. Here we refer to the people we interviewed as “Service Providers” (SPs) and their overall organizations which they represent as “Provider Organizations” (POs).

### The current study

Some authors have argued that there is a distinct difference between contracting out relationships where the public sector specifies the solutions, and true PPPs which should involve joint decision-making to ensure effective outcomes for all partners (Klijn & Teisman,, 2000). However, among the many conceptualizations of PPPs described in the literature, concepts such as shared objectives, cooperation, synergy, shared risk taking and mutual benefit are borne out with great variation in practice (Roehrich et al., 2014). A recent review of the health PPP literature (Roehrich et al., 2014) concluded that there are still evidence gaps regarding the function and benefits of PPPs. We therefore aim to address this by 1) evaluating the satisfaction of (private) SPs with their participation in delivery of the GHaW programme and 2) considering what the GHaW SPs’ experiences might imply for using PPPs in health promotion. This study has ethics approval from the University of Sydney Human Research Ethics Committee #2014/1014.

## Methods

### The GHaW programme

In 2015, the NSW government implemented a workplace health programme to encourage businesses across the state to participate in a workplace intervention to improve the health of workers. The programme included a brief health check (BHC) for employees to know their own health status, a workforce health summary for employers where more than 50 employees provided data, assistance to develop an action plan (selection of health priority and details of what will be implemented to address it) and a small financial subsidy towards implementing health promotion in the workplace. The programme was tailored to focus workplace health promotion on government-identified priority areas (i.e., smoking cessation, improving physical activity, and healthy diets, healthy weight or active travel). The programme was delivered either through a 1) do-it-yourself online method or, 2) with the help of a SP or 3) through a combination approach (e.g., online health checks with SP assisting the action plan development).

### GHaW SP role

Where businesses opted for SP support, the SP’s role encompassed provision of expert advice and support through a five-step system of gaining leadership support, assessing the workplace needs, identifying a priority health issue, developing a plan and evaluating the intervention. SPs also conducted Brief Health Check (BHC)s for individual workers. POs were renumerated according to completion of programme steps with rates taking account of business size, thereby acknowledging the complexity of planning interventions for larger businesses. A guide for the number of hours and type of interaction (face to face, phone, email) for each of the five steps was provided. Delivery of intervention activities was not funded, however providers were permitted to offer additional for-profit services where the content and fee was pre-approved. The SP role for delivering BHCs involved supporting businesses to promote the service, schedule participants, conduct the health check, make referrals where appropriate and securely transfer collected health data. Providers were remunerated per health check completed with rates determined by the expected effort to promote and schedule checks, geographical region, and total volume of health checks delivered. Further detail regarding the GHaW programme may be found elsewhere (Crane, Bohn-Goldbaum et al., 2019; Khanal et al., 2017).

### Study design and recruitment

Qualitative in-depth interviews were undertaken with SPs from the POs involved in GHaW at the time of the evaluation in 2016 (n = 10 people from 9 organizations; 1 refusal (no reason given)) (Khanal et al., 2017). The GHaW programme manager supplied contact details for current POs. POs were contacted by independent university researchers by email and forwarded a participant information statement and a consent form. The PO contact decided the best staff member to be interviewed. The primary account manager(s) responsible for GHaW and/or who carried out most of the GHaW interactions with businesses was interviewed, and for one PO two SPs (SP6-A and SP6-B) were interviewed simultaneously. Email contact was followed by phone contact and interviewees either sent an electronic copy of a signed consent form by return email or provided verbal consent and the signed form at interview. We note that those invited were a subset of the 15 POs contracted at the beginning of the programme and the remainder had withdrawn from the programme at the time of study recruitment. The researchers were only given the details of the current POs and therefore could not contact those which no longer were associated with the programme. Participants were informed that the interviewers were independent of the GHaW programme with an interest in health programme evaluation.

### In-depth interviews

Interviews were conducted face-to-face (n = 9 participants) at the SP’s workplace or by phone (n = 1) and were digitally recorded with permission. The semi-structured interview guide was developed by two researchers with knowledge of WHP (AG & MC) in consultation with the GHaW programme manager to ensure understanding of programme components and it addressed their evaluation needs. Questions covered the educational and occupational background of the interviewee and PO staff delivering the intervention; how the PO became involved with GHaW and the SP’s impressions and attractions of the programme (e.g., What do you think of GHaW concept?); engagement of workplaces in GHaW (e.g., Please describe workplaces you have had contact with (industries, size, type of workers)?); their experience of each stage of the GHaW programme cycle (e.g., What do you think of the registration process and resources provided to business to “get the ball rolling”?) (Khanal et al., 2017); and perceptions of the impact of GHaW on workplaces (e.g., Did you perceive a change in workplace climate and culture in terms of health matters in any of the workplaces?) Interviews were conducted by MC, AG and EG, all experienced qualitative interviewers and health researchers, lasting between 46 and 87 minutes each. All interviewers were female and have an interest in workplace health programmes for the prevention of lifestyle-related non-communicable diseases.

### Analysis

Interview recordings were transcribed verbatim and managed in NVIVO (QSR International Pty Ltd, 2015). We conducted the analysis according to the stages described by Braun and Clarke (2006). The lead researcher (AG) along with two other researchers (EG, MC) familiarized themselves with the interviews through listening to the recordings and correcting the transcripts (stage 1). AG then conducted a first-level analysis (stage 2) using line-by-line coding using inductive approach with codes relevant to understanding interviewee’s experiences with GHaW. Initial codes were discussed with three other researchers (EG, MC, MT) to check coding and interpretation. AG iteratively developed conceptual themes through grouping initial codes together under higher level headings, testing these against the data (stage 3) in terms of internal coherence and meaning given the evaluation questions. AG and MT continued reviewing themes and sub-themes to see whether they were reflected across the whole dataset and refining their interpretation (stage 4), until no new themes emerged (thematic saturation) (Saunders et al., 2018). The final interpretation of themes was discussed with all authors. Participant feedback on findings was not sought as participant details were not retained.

## Results

The interviewees’ (n = 10) professional backgrounds spanned a range including nursing, personal trainer, psychology, occupational therapy, and nutrition; they were the account managers for GHaW for their organization and/or worked directly with GHaW workplaces. Although the degree of hands-on implementation of GHaW varied across the interviewees, all had good knowledge their organization’s experience with GHaW. All except one interviewee was female.

Our analysis of the interviews with SPs generated three main themes about their experience implementing GHaW. These were alignment, misalignment and the anticipated benefit or “value-add” of GHaW. The theme of alignment broadly reflected how SPs felt their core work and vocational goals aligned with those of the programme hence confirming their suitability to implement the programme. The misalignment theme describes where operational aspects and expectations differed between the programme and SPs, and their impact on implementation. Finally, the value of GHaW to both the SP, their PO and the participating workplaces was key to understanding the implementation experience of the SPs. Each of these themes and their subthemes are described below with illustrative quotes from our interviewees. The implications of the findings for the partnership are then explored in the discussion.

### Theme 1: alignment

A major theme that described the SPs’ experience of GHaW was the “alignment” between the GHaW programme and the work and people of the POs. The SPs saw their own motives, core values and skills/expertise as aligned with the aims of the GHaW programme and its potential to positively impact on businesses and workers.

#### Purpose, motives and core values

Firstly, the SPs felt that it was appropriate for the government to be supporting businesses to encourage workers to improve their health. The interviewees felt strongly that the government should intervene and that GHaW could make a difference through providing health programmes in the workplace setting, especially where businesses were unlikely to be able to provide these services themselves.

***SP2***: *I thought that it was really good that the government was doing something about it in terms of providing assistance in terms of helping workplaces to set something up a little bit more formally.*

***SP3***: *I think it’s a really positive initiative to get people starting to think and to provide them with somebody to help them and to guide them through the process. I think it’s really positive to provide them with some resources and tools to work towards it.*

There was also alignment between the GHaW programme content and the core values of SPs. For example:

***SP3***: *Well, I guess if you’re a health professional who’s chosen to work in corporate health you have to have a very strong belief in value of work to somebody’s well-being … It’s absolutely something that’s of our benefit in selling this.*

***SP1***: *I think I am very good at selling something I am passionate about. So [GHaW] have a lot of events, so I get to speak to a lot of people at events as well.*

Strong alignment of the programme with the values of individual SPs meant they were committed to making the programme a success and as such would often go beyond for what they were remunerated. For example, some SPs mentioned they would often travel to places outside of their geographic range, spend time on tasks which would not be covered by the funding arrangement, exploit their own contact databases in order to promote the programme and pursue clients even when the probability that it would be financially rewarding was questionable:

***SP4***: *As a service provider, we don’t get paid that much, we just get paid for the health checks, the signing up et cetera, you don’t get paid all those hours of phone calling, negotiating, calling on them, helping them out, so there’s a lot of push and a lot of work.*

***Interviewer***: *So, what motivates you to do it?*

***SP4***: … well I’m a nurse, you know. Health promotion is part of my job.

***SP2***: *[referring to out of standard hours work] you’d understand our staff have families, personal lives too, to get back to. But we want to see this programme … through and see it successful.*

The concordance of the GHaW purpose and aims with SPs’ field of workplace health promotion gave meaning to their involvement in the programme, and aligned in principle with their vocational goals. It engendered interest and enthusiasm for the programme and a sense of shared purpose.

***SP5***: *we will try to set [a workplace] up with a free programme that they can run, you know, ‘cause the outcome, or anything that my fellow coach and myself want is for the workplace to actually continue on doing something.*

***SP2***: *I thought [GHaW] was an initiative that could be reached by everyone so I thought that was good and that’s what I found interesting.*

#### Skills/expertise

Our interviewees also felt they had the right skills, and employed professionals who were passionate about, well suited to and experienced in delivering lifestyle-related chronic disease prevention programmes.

***SP1***: *so I thought [GHaW] was an opportunity to get my hands in, because I’m very passionate about health and fitness and I thought in a corporate space it is an exciting area.*

***SP3***: *They’re all physios or OTs that are going. .They’ve all got their training and their background. It’s just a matter about skilling them in terms of what actually Get Healthy at Work is and the parameters of that before they go out.*

For a couple of the POs targeting particular health conditions (e.g., workplace injury), the more general well-being and lifestyle risk factor approach of GHaW provided vocationally relevant opportunities to use and expand staff skill sets.

***SP3***: *It speaks to the OT side of me, I suppose, having worked in a rehab hospital with lots of people who’ve had diabetes, heart attack, stroke. It was something that was interesting to me and something that was a blast from the past for me, so I enjoyed getting involved in it and selling it.*

***SP6-A***: *We recently changed our constitution as an organization so that we’re working more in the preventative space and this is something that we see as an opportunity to do that.*

### Theme 2: misalignment

Co-existing with the sense of alignment described above, our interviewees described a number of misalignments occurring along different axes, placing the programme, the SPs, and/or the workplaces, at odds. Misalignment manifested in subthemes of strategic, operational and outcome aspects of the programme.

#### Misalignment on programme strategy

GHaW was designed to support workplaces to work through a cycle of steps to build capacity in delivering WHP and change the culture and environment to be more health supporting. SPs felt that businesses’ perceptions of the programme were misaligned with this capacity building strategy and that businesses perceived the SP’s role as running the programme rather than facilitating the development of a programme by the workplace itself. Consequently, SPs reported that the initial enthusiasm of a business for the programme faded once they realized the required commitment which presented a serious challenge to SPs in their implementation role.

***SP7***: *And unfortunately people see okay, there is a service provider involved, they’re going to do it for me. And this is a free programme and they’re as hands off as possible. So these people are unlikely to progress through the programme very far.*

***SP6-B***: *I think from those smaller businesses that are really wanting the programme, obviously they’ve looked into it, they’ve just been overwhelmed, just from the get-go. And they’re the ones that are pulling out, they’re not contacting us.*

There was an element of complicity on the part of SPs, which may have reinforced this perspective: although they realized it was not in keeping with the GHaW approach, SPs reported taking on tasks that the business was allocated in order to progress them through the programme.

***SP2***: *There’s been very few that have the time I think, or allow themselves the time to explore the website and use those online resources. … but most of them rely heavily on us attaching them to emails, directing them to where it is, adding a link, I guess that spoon fed sort of notion is and because as a service provider our role is to facilitate the rolling of that programme for them, I guess their expectation is that we fulfil that …*

Misalignment of programme strategy between GHaW/SPs and businesses was consequential for the partnership insofar as SPs would take on work they were not paid for and/or the capacity-building for businesses for which GHaW was designed was not achieved.

#### Misalignment of programme cycle and business processes

All of the SPs described the importance of momentum in achieving GHaW programme goals and yielding satisfactory outcomes for them as SPs and their POs, as well as for the businesses registered in the programme. Momentum meant that businesses remained engaged with and moving through the programme and SPs could reach the milestones for which they were paid. Key ingredients to keep businesses moving through the GHaW programme cycle according to our interviewees were workplace timing and readiness for such a programme, continuity of key personnel and GHaW processes functioning to facilitate the smooth running of the programme. The absence or misfiring of any of these key factors undercut momentum and progress. For example, the misalignment of internal operational rhythms of workplaces with the GHaW programme cycle led to a break in momentum which in turn led to discontinuation or presented as an insurmountable barrier to commence.

***SP1***: *I found at the end of the financial year, that was the worst, because people that got like, other commitments and the Get Healthy at Work programme is probably not a commitment at the end of financial year, you know …*

A number of the SPs also mentioned the difficulty with larger business was the delay entailed when approval for the programme required agreement across several layers of management.

***SP8***: *But when we get to a larger organization*, *we're talking to one contact, but with various locations, a group of maybe six or ten, and then on top of that, there’s another level there that we need to get all 30 people on board, to be able to run the programme further.*

Stable and continuing leadership for GHaW within the business and progress through the GHaW cycle were bi-directionally linked: if leadership within the business changed, continuation in the programme was uncertain. Similarly, delays in the GHaW program’s response to the workplaces increased the likelihood that the personnel or appetite for implementing a WHP might change and the momentum in the business was lost.

***SP8***: *By the time they make up their mind, and I’m just giving an example, this HR person could have left the organization*,*or moved on to a different division.*

Finally, a couple of SPs said that the GHaW programme components could prompt businesses to withdraw which could derail the programme completely:

***SP2:*** *we had a lot of business that were held up at that statement of commitment, because … they had this idea that it was a legal document and if they signed it, it exposed them to all sorts of obligations … it’s why we haven’t actually seen the amount of businesses we should have seen already come through the whole programme. …*

***SP6-A***: *… the current structure [of GHaW] means there’s steps in the process that are points where you can lose motivation along each path, each step of the path.*

### Theme 3: potential vs realized benefits

Another theme throughout the SP interviews which sheds light on how they experienced the GHaW programme was the question of the added value or benefits which the programme could provide to the participants. The theme centred on three main sub-themes which were the benefits to POs of GHaW as a business proposition, the benefits to businesses as an enhanced WHP initiative and, in terms of reputation, the value of the programme for both POs and businesses.

#### GHaW as a business proposition

One of the incentives for POs to participate in GHaW was the potential for bringing new business to their company. The SPs reported that along with the attraction of working to improve workplace health in the community (see Alignment, above) GHaW could dually serve their profit motive.

***SP3***: *It really is a feel-good service for us. It also opens a few doors for us in terms of lifting our profile with corporate customers as well because a lot of the employers that are becoming involved are potentially corporate customers.*

However, the potential seemed to work unevenly. For example, the point system which earned businesses funds to purchase the services of the SPs relied upon businesses reaching certain milestones, milestones which were frequently not reached.

***SP6-A***: *As I’ve said we do run educational sessions within workplaces but it’s not our core business. We have that in our toolkit, but we’re just not even getting to the point where we’re delivering an action plan so we can offer those services.*

Whilst there was motivation beyond generating new business (see section Alignment), the profit motive was reported to have curtailed some investment in GHaW because the accountability structures and processes within the PO demanded it.

***SP3***: *It’s an expensive thing for us to run, it doesn’t reimburse itself. We have to hope that the benefit of it will be that we’re seen as a market leader and value add service as well because it’s not going to come back to us financially through Get Healthy ever.*

***SP5***: *so if we go to [place] with kilometres and travel if I am paying on my staff, we do not take much home from that at all. So the incentive for us to sell [GHaW] is a lot lower than to sell ours.*

Payment processes sometimes did not fit with the reality of how the relationships with business worked which also created tension between trying to implement the programme well and maintaining financial viability.

***SP1***: *I could spend 2 hours organizing health checks, getting other exercise physiologists on board and then trying to co-ordinate the availabilities and what have you, but that is not paid for [by GHAW]. That is paid by my manager.*

SPs also reported a business could have number of people register for BHCs but only a small proportion of registrants attend. However, POs were only paid for the number of BHCs actually performed. Travel to business locations was also not covered which meant that SPs became reluctant to take on clients which involved travel [subsequently both no-show and travel payments were added to the program]. SPs felt that businesses would view this reluctance as a shortcoming of the service they provided as they assumed that the SPs were covered for the travel irrespective of the turnout.

Thus PO and their SPs’ expectations of GHaW for business development and profit-making were misaligned with GHaW in practice as some tasks necessary for conducting the programme did not attract remuneration and the rewards system was contingent on rarely met milestones. It appears that neither SPs nor businesses were fully aware of all these programme details when joining the programme, nor did they have the opportunity to contribute at the design stage.

#### GHaW as value-add in workplace health for workplaces

In general, SPs felt that the programme could be helpful to workplaces, but not in all cases and not all components. In particular the GHaW key component of BHCs were something which some SPs felt had limited appeal. The format used by GHaW was often discussed in comparison to other “health check” procedures SPs had used and there was some sense that they did not meet SPs’ or businesses’ expectations of what would be informative and satisfy participants. In particular it was felt that the lack of objective assessment (e.g., taking blood samples or measuring blood pressure) reduced the perceived value to workers and undermined the validity of the check.

***SP9***: *We’ve had a mixed response [to BHCs]. A lot of them is “great, overall”. We’ve had some, whether it’s been the workers, and the companies itself going, “It’s just a waist measurement.” When they’ve done pre-employment medicals before, they incorporate absolutely everything.*

On the other hand, some found BHCs useful for identifying the salient issues for the workplaces. One SP, in particular, felt that it was superior to more invasive health checks because workers would not fear their privacy may be violated with this less invasive style of assessment.

***SP8***: *But I do understand because in their marketing they’ve said there’s no needles, there’s no feedback to say well we’re testing for drugs or we’re testing for something else other than that*.

SPs also had positive and negative views about the benefits to workplaces of the programme itself. For example, for larger businesses that might have their own dedicated health and well-being resources and programmes, the low-intensity GHaW programme would not add much value. For smaller businesses, however, GHaW was seen as providing access to assistance which would be otherwise unaffordable and allow them to act upon plans that they may have had but not been able to previously resource.

***SP6-B***: *And because those larger businesses do have their own workers’ health officers, they tend to look at the programme and then just think “oh we can do this ourselves”.*

The cost-free nature of GHaW was mostly seen by the SPs as an advantage of the programme as it improved accessibility, especially in the context of small businesses which would otherwise not have access to support for workplace health programmes. However, other SP perceptions were that GHaW wasn’t valued if it was free or viewed suspiciously as the “hook” to trick businesses into signing up for future expenses:

***SP7:*** *Because it’s a free programme, they don’t seem to value that we’re actually here to do some work.*

***SP5***: *What is it then going to be? What else do we have to pay for?*

Further, SPs felt workplaces could not see the value of improving worker health, and/or it would always come second to core business:

***SP7***: *This is often not seen as a business priority. Even though we say ourselves here that, “For every dollar you spend, 3 USD-$7 would come back in the health of your staff.” That doesn’t mean as much as it should to many businesses.*

A number of the SPs felt the GHaW resources were helpful for businesses, and often forwarded the materials to businesses as part of their support role. Sometimes they adapted these resources or supplemented them with their own.

***SP7***: *I think there’s nuggets of information in there of info that people use. I don’t know that people would use them as they are. I think that people would take out the information, put them in their own email, their own letterhead and send it out*

The value-add to workplaces also interacts with the misalignment on the GHaW programme strategy described in the previous section because SPs argued that the effort which workplaces were required to expend devalued the programme for many.

#### Reputational reward and risk

SPs felt the reputational opportunities associated with GHaW were two-fold: first having a contract with the state government health department brought prestige in that their POs could be entrusted to deliver services as part of a large-scale government programme; therefore being associated with GHaW (at least in the abstract) would enhance their reputation as workplace health POs. Some POs used this as a marketing tool to increase their exposure.

***SP8***: *For our clients I think they see us as being a reputable service provider, to be working under the government.*

***SP7***: *But it’s good to be seen as a company, … to be aligned with the government and supporting peoples’ health at a … state level*

Second, according to our interviewees, not only did GHaW provide an opportunity for POs to enhance their reputation, it was an opportunity for businesses to cultivate capital with their own employees in that it could not only potentially improve their workers’ health but also signal an interest in their welfare.

***SP7***: *But I sell the idea of the brief health check as more a way that the staff can get on board with their own health … That it’s a way for the company to show the staff that they’re interested in their health.*

At the same time, SPs felt that some GHaW processes compromised their business professionalism and risked their reputation. For example, the SPs reported that many workplaces found the GHaW portal difficult to navigate and would ask the SP for advice, yet SPs lacked access to the businesses’ portal. In other cases, SPs did not have direct access to BHC data and therefore had to rely on the business forwarding this information in order to assist in formulating a target risk factor and action plan. Some SPs also felt that the lack of progress of their client businesses through GHaW reflected poorly on them as facilitators:

***SP5***: *I think it also comes across that we are confused, … when the websites are not working, like we couldn’t make changes, it took us like 3 weeks for [GHaW] to get back to us to be able to make it, so it’s, whereas when it’s our product, we are a lot more confident, we can do this, this is what is going to happen*

***SP9***: *Sometimes, the client turns around and says, “What you mean you don’t have a copy of the report?” We turn around and say, “Look, can you send us the report that you’ve received?” It just loses a little bit of that professionalism because we’ve been doing everything in the background for them …*

***SP3***: *It was just sidelined, it’s just constantly sidelined and that’s not good from a couple of perspectives. One, it ruins our reputation but, two, it’s companies that are actually thinking about doing something positive for their workers and if we’re not helping them to do that and we’re making it difficult for them to do that …*

Thus the reputational value-add of association with a state government health programme could be undermined by the programme implementation process where procedures were suboptimal and hindered the ability of SPs to support businesses to progress through the programme.

## Discussion

Our study is one of the few to examine a PPP for health promotion from the perspective of the private sector partner. We analysed interviews with workplace health programme SPs in the GHaW programme and characterized their experiences through three main themes: alignment, misalignment and the benefit or value-add of GHaW to them. We now reflect on these themes and connect to previous research on workplace health programmes as well as the PPP literature, and note the implications for future practice.

Despite the apparent alignment of the objectives of the POs and the government’s GHaW programme, according to our interviewees a number of factors undermined SPs achieving the benefits of partnering on the programme they had anticipated from being contracted to deliver the services. For example, the expected synergy between the government’s resources and the skills and expertise of the SPs was largely not realized as the efforts of SPs proved insufficient to support and encourage many businesses through the programme steps. Making GHaW free addressed one of the major barriers to WHP implementation in Australia (Taylor et al., 2016), especially for small to medium-sized business, but that also led to it not being valued by workplaces and they seemed to be at varied stages of readiness to implement a WHP . With payments to POs contingent upon completion of the specified components, registered workplaces not ready to prioritize or commit to the programme contributed to a poor rate of completion and dissatisfaction on the part of POs and their SPs whose success and financial viability depended on the businesses progressing through the programme. The wide targeting and prima facie free intervention perhaps attracted workplaces unsuited to the programme, but with which SPs nonetheless invested time in engagement and follow up. Narrower targeting, or assessment of readiness to engage prior to registering to participate may be mechanisms which could enhance matching of workplaces to the programme.

Within the businesses themselves, other factors such as organizational capacity (Durlak & DuPre, 2008) impacted successful implementation of WHP and their capacity for sustainability (Schell et al., 2013). Leadership (Corbin et al., 2016) and having workplace champions (Waterworth et al., 2016) to carry implementation forward are some factors cited as promoting successful implementation and outcomes of WHPs . In our interviews, the SPs reported that if participation of a business was driven by particular people (i.e., champions) who subsequently left, there were often problems with subsequent programme progression. Whilst there was agreement that having an active key contact was helpful, it also left the programme vulnerable to not progressing through the cycle if circumstances changed. Hence ongoing adaptation to transfer knowledge within the workplace may be necessary or devolve ownership to a broader group (Joss et al., 2017) to ensure programmes are maintained when leadership or contacts change. From the programme side, ensuring a support system where processes are not unnecessarily complex in ways which may delay implementation will enable more effective implementation (Durlak & DuPre, 2008).

The tension created between the aim of the programme to build capacity for workplaces to run their own programmes and the for-profit nature of POs also challenged implementation. POs had expected to substantially broaden their client base and sale of services through the programme reflecting the notion of synergy in partnership whereby it facilitates access to something which would not be available to each partner alone (Corbin et al., 2016). However, when those expectations went unmet, the SPs and their POs became less inclined to invest effort in GHaW and a feeling of antagony (rather than synergy) predominated where a partner believes they achieve less by working in partnership than on their own (Corbin & Mittelmark, 2008). While partnerships for service provision may be one solution to sustainability it remains unclear whether they can compensate for limited funding (Shelton & Lee, 2019); a PPP model may not meet private sector for-profit motives if paid components do not compensate for the investment of time and resources.

An important dynamic between POs and businesses was that the latter had the power to withdraw from GHaW when they believed their interests were not being served, and could do so with little risk of impact or consequence from the programme. Our interviewees offered many reasons why programme withdrawal occurred, from differing expectations of the SP role and hesitancy about the amount of work involved on the business’s part, to problems with the operation and administration components of the programme. By contrast, SPs reported that they and their organization bore a number of risks from businesses withdrawing, including time invested to engage the businesses in the programme, reputational risk of being ineffective and the dependence of remuneration for service delivery upon businesses meeting thresholds and milestones. Given the importance of sharing and/or shifting of risk in developing PPPs (Klijn & Teisman, 2000), the perceived imbalance in consequences between the POs and workplaces in failure to complete programme components undermined the functioning of the partnership. Previous research argues that resolving issues of governance is an ongoing task within PPPs and therefore providing opportunities to collaboratively address tensions and conflicts is key to achieving synergy (Klijn & Teisman, 2000).

It is important to note that whilst we have framed GHaW as a form of partnership, several authors have highlighted that lack of shared decision making on agenda-setting, goals and strategies places these arrangements more appropriately as “public-private interactions” (Johnston & Finegood, 2015) or merely financial or business contractual relationships (Klijn & Teisman, 2000). Whether the GHaW programme in its broadest sense constitutes a partnership or cross-sector interaction is debatable based on the data presented here. A number of the features of the latter include that the relationship is largely transactional, there is organizational independence, the goals have been determined by one partner and there is a narrow scope of activities. However, it also appears to operate in a more partnership-like manner in that, for example, there is authentic trust, the goals of partners are central to the mission and there is contribution of resources from both GHaW and POs (i.e., GHaW programme portal, materials, funding from government and SP skills, expertise, intervention techniques and client-base) towards a common goal. Regardless, it is clear that features of PPPs previously identified as relevant to good functioning [or to good success] are also relevant in framing the SP experience. Further, the PPP approach may be becoming more common as a vehicle for health promotion interventions delivered to the population and hence examining the private PO experience with a view to sustainability is important if large scale interventions are to have population impact.

### Strengths and limitations

Our research described the experiences of SPs, which are often missed in the evaluation of large-scale complex interventions (Crane, Bauman et al., 2019). Case studies of PPPs, especially in health promotion, appear infrequently in the literature but are important for assessing their value to all partners. Our study included SPs who were delivering the GHaW programme at the time of the evaluation, so we can confidently rely on their views as demonstrating the experience of SPs from this perspective, but several POs had already exited the programme so we have no data on their views. This current analysis is limited to the SPs, who represent only one component of GHaW implementation; we do not have the views of the government partners (the resource team) or the workplaces involved in the implementation of the programme for comparison. Given that different partners within the same partnership can perceive the success or failure of activities differently (Corbin et al., 2016), it would be instructive to compare these data with the perceptions of the GHaW programme managers. Conclusions therefore may only be drawn for the perceptions of this group rather than the delivery system as a whole. Moreover, the interviews for this analysis describe the experiences of the SPs rather than partnership *per se* and therefore the commentary related to partnership is interpretive rather than deliberate reflections on the concept of partnership in GHaW. Therefore, the SPs may have more or different opinions about the arrangement which are not contained in this dataset.

## Conclusion

Our findings suggest that the workplace is a complex environment into which to bring a health promotion intervention using private POs, especially if a single, albeit flexible, programme is proposed to be implemented across a range of business sizes and industries. The need for POs to achieve financial benefits and the misalignment between expectations of businesses and the programme goals challenged sustained involvement of POs and businesses. Although private industry may be contracted to implement a government intervention, evaluating the experience of SPs in the GHaW programme reveals practical constraints, which have direct consequences for programme outcomes, particularly for programme sustainability. A PPP model is atypical for WHP interventions which usually seek to achieve organizational change through persuasion and commitment of resources from private industry, rather than encourage change by providing resources to them (Johnston & Finegood, 2015; Roehrich et al., 2014). Careful consideration must be given to the functioning of cross-sector engagement and partnering in practice in order to ensure public health goals are met and that the model is viable for all partners to be sustainable.

## Data Availability

Due to the nature of this research, participants of this study did not agree for their data to be shared publicly, so supporting data is not available.

## References

[cit0001] Anderson, L. M., Quinn, T. A., Glanz, K., Ramirez, G., Kahwati, L. C., Johnson, D. B., Buchanan, L. R., Archer, W. R., Chattopadhyay, S., Kalra, G. P., & Katz, D. L (2009). The effectiveness of worksite nutrition and physical activity interventions for controlling employee overweight and obesity: A systematic review. *American Journal of Preventive Medicine*, 37(4), 340–13. 10.1016/j.amepre.2009.07.003 19765507

[cit0002] Anger, W. K., Elliot, D. L., Bodner, T., Olson, R., Rohlman, D. S., Truxillo, D. M., Kuehl, K. S., Hammer, L. B., & Montgomery, D. (2015). Effectiveness of total worker health interventions. *Journal of Occupational Health Psychology*, 20(2), 226. 10.1037/a0038340 25528687

[cit0003] Astrella, J. A. (2017). Return on investment: Evaluating the evidence regarding financial outcomes of workplace wellness programs. *JONA: The Journal of Nursing Administration*, 47(7/8), 379–383. 10.1097/NNA.0000000000000499 28727623

[cit0004] Batley, R., & McLoughlin, C. (2010). Engagement with non-state service providers in fragile states: Reconciling state-building and service delivery. *Development Policy Review*, 28(2), 131–154. 10.1111/j.1467-7679.2010.00478.x

[cit0005] Blackford, K., Jancey, J., Lee, A. H., James, A. P., Waddell, T., & Howat, P. (2016). Home-based lifestyle intervention for rural adults improves metabolic syndrome parameters and cardiovascular risk factors: A randomised controlled trial. *Preventive Medicine*, 89, 15–22. 10.1016/j.ypmed.2016.05.012 27196148

[cit0006] Braun, V., & Clarke, V. (2006). Using thematic analysis in psychology. *Qualitative Research in Psychology*, 3(2), 77–101. 10.1191/1478088706qp063oa

[cit0007] Cahalin, L. P., Kaminsky, L., Lavie, C. J., Briggs, P., Cahalin, B. L., Myers, J., Forman, D. E., Patel, M. J., Pinkstaff, S. O., & Arena, R. (2015). Development and implementation of worksite health and wellness programs: A focus on non-communicable disease. *Progress in Cardiovascular Diseases*, 58(1), 94–101. 10.1016/j.pcad.2015.04.001 25936908

[cit0008] Chau, J. (2009). *Evidence module: Workplace physical activity and nutrition interventions*. Physical Activity Nutrition and Obesity Research Group, University of Sydney.

[cit0009] Corbin, J. H., Jones, J., & Barry, M. M. (2016). What makes intersectoral partnerships for health promotion work? A review of the international literature. *Health Promotion International*, 33(1), 4–26. 10.1093/heapro/daw061 PMC5914378 27506627

[cit0010] Corbin, J. H., & Mittelmark, M. B. (2008). Partnership lessons from the global programme for health promotion effectiveness: A case study. *Health Promotion International*, 23(4), 365–371. 10.1093/heapro/dan029 18835888

[cit0011] Crane, M., Bauman, A., Lloyd, B., McGill, B., Rissel, C., & Grunseit, A. (2019). Applying pragmatic approaches to complex program evaluation: A case study of implementation of the New South Wales Get Healthy at Work program. *Health Promotion Journal of Australia*, 30(3), 422. 10.1002/hpja.239 30860630

[cit0012] Crane, M., Bohn-Goldbaum, E., Lloyd, B., Rissel, C., Bauman, A., Indig, D., Khanal, S., & Grunseit, A. (2019). Evaluation of Get Healthy at Work, a state-wide workplace health promotion program in Australia. *BMC Public Health*, 19(1), 183. 10.1186/s12889-019-6493-y 30760237 PMC6373144

[cit0013] Durand, M. A., Petticrew, M., Goulding, L., Eastmure, E., Knai, C., & Mays, N. (2015). An evaluation of the Public Health Responsibility Deal: Informants’ experiences and views of the development, implementation and achievements of a pledge-based, public–private partnership to improve population health in England. *Health Policy*, 119(11), 1506–1514. 10.1016/j.healthpol.2015.08.013 26433565

[cit0014] Durlak, J. A., & DuPre, E. P. (2008). Implementation matters: A review of research on the influence of implementation on program outcomes and the factors affecting implementation. *American Journal of Community Psychology*, 41(3–4), 327. 10.1007/s10464-008-9165-0 18322790

[cit0015] Elliott, T., Trevena, H., Sacks, G., Dunford, E., Martin, J., Webster, J., Swinburn, B., Moodie, A. R., & Neal, B. C. (2014). A systematic interim assessment of the Australian Government’s Food and Health Dialogue. *Medical Journal of Australia*, 200(2), 92–95. 10.5694/mja13.11240 24484111

[cit0016] Klijn, E.H & Teisman, G.R. (2000). Chapter 5 Governing public-private partnerships. In S. P. Osbourne (Ed.), *Public-private partnerships. Theory and practice in international perspective,*84-102. Routledge.

[cit0017] Goetzel, R. Z., Shechter, D., Ozminkowski, R. J., Marmet, P. F., Tabrizi, M. J., & Roemer, E. C. (2007). Promising practices in employer health and productivity management efforts: Findings from a benchmarking study. *Journal of Occupational and Environmental Medicine*, 49(2), 111–130. 10.1097/JOM.0b013e31802ec6a3 17293753

[cit0018] Grossmeier, J., Fabius, R., Flynn, J. P., Noeldner, S. P., Fabius, D., Goetzel, R. Z., & Anderson, D. R. (2016). Linking workplace health promotion best practices and organizational financial performance: Tracking market performance of companies with highest scores on the HERO scorecard. *Journal of Occupational and Environmental Medicine*, 58(1), 16–23. 10.1097/JOM.0000000000000631 26716844

[cit0019] Grunseit, A. C., Rowbotham, S., Pescud, M., Indig, D., & Wutzke, S. (2016). Beyond fun runs and fruit bowls: An evaluation of the meso-level processes that shaped the Australian Healthy Workers Initiative. *Health Promotion Journal of Australia*, 27(3), 251–258. 10.1071/HE16049 27745572

[cit0020] Hardy, L. L., King, L., Kelly, B., Farrell, L., & Howlett, S. (2010). Munch and Move: Evaluation of a preschool healthy eating and movement skill program. *International Journal of Behavioral Nutrition and Physical Activity*, 7(1), 80. 10.1186/1479-5868-7-80 PMC2988057 21047434

[cit0021] Hector, D., & St George, A. (2013). *Scoping review for the NSW Get Healthy@Work Organisational Support Service: A component of the NSW Healthy Workers Initiative*. Physical Activity Nutrition & Obesity Research Group, University of Sydney.

[cit0022] Johnston, L. M., & Finegood, D. T. (2015). Cross-sector partnerships and public health: Challenges and opportunities for addressing obesity and noncommunicable diseases through engagement with the private sector. *Annual Review of Public Health*, 36(1), 255–271. 10.1146/annurev-publhealth-031914-122802 25581149

[cit0023] Joss, N., Dupré-Husser, E., Cooklin, A., & Oldenburg, B. (2017). The emergence of integrated approaches to worker health, safety and wellbeing in Australia. *Australian Journal of Primary Health*, 23(2), 154–161. 10.1071/PY16065 27604852

[cit0024] Khanal, S., Lloyd, B., Rissel, C., Portors, C., Grunseit, A., Indig, D., Ibrahim, I., & McElduff, S. (2017). Evaluation of the implementation of Get Healthy at Work, a workplace health promotion program in New South Wales, Australia. *Health Promotion Journal of Australia*, 27(3), 243–250. 10.1071/HE16039 27816068

[cit0025] King, E., Grunseit, A., O’Hara, B., & Bauman, A. (2013). Evaluating the effectiveness of an Australian obesity mass-media campaign: How did the ‘Measure-Up’ campaign measure up in New South Wales? *Health Education Research*, 28(6), 1029–1039. 10.1093/her/cyt084 23962490

[cit0026] Kite, J., Gale, J., Grunseit, A., Bellew, W., Li, V., Lloyd, B., Maxwell, M., Vineburg, J., & Bauman, A. (2018). Impact of the Make Healthy Normal mass media campaign (Phase 1) on knowledge, attitudes and behaviours: A cohort study. *Australian and New Zealand Journal of Public Health*, 42(3), 269–276. 10.1111/1753-6405.12779 29644787

[cit0027] Marteau, T. M., Hollands, G. J., & Fletcher, P. C. (2012). Changing human behavior to prevent disease: The importance of targeting automatic processes. *Science*, 337(6101), 1492–1495. 10.1126/science.1226918 22997327

[cit0028] Muka, T., Imo, D., Jaspers, L., Colpani, V., Chaker, L., Van der Lee, S. J., Mendis, S., Chowdhury, R., Bramer, W. M., Falla, A., Pazoki, R., & Franco, O. H. (2015). The global impact of non-communicable diseases on healthcare spending and national income: A systematic review. *European Journal of Epidemiology*, 30(4), 251–277. 10.1007/s10654-014-9984-2 25595318

[cit0029] O’Hara, B. J., Grunseit, A., Phongsavan, P., Bellew, W., Briggs, M., & Bauman, A. E. (2016). Impact of the swap it, don’t stop it Australian national mass media campaign on promoting small changes to lifestyle behaviors. *Journal of Health Communication*, 21(12), 1276–1285. 10.1080/10810730.2016.1245803 27892841

[cit0030] Osborne, S. (2000). *Public private partnerships. Theory and practice in international perspectives*. Routledge.

[cit0031] QSR International Pty Ltd. (2018) NVivo (Version 12), https://www.qsrinternational.com/nvivo-qualitative-data-analysis-software/home.

[cit0032] Roehrich, J. K., Lewis, M. A., & George, G. (2014). Are public–private partnerships a healthy option? A systematic literature review. *Social Science & Medicine*, 113, 110–119. 10.1016/j.socscimed.2014.03.037 24861412

[cit0033] Saunders, B., Sim, J., Kingstone, T., Baker, S., Waterfield, J., Bartlam, B., Burroughs, H., & Jinks, C. (2018). Saturation in qualitative research: Exploring its conceptualization and operationalization. *Quality & Quantity*, 52(4), 1893–1907. 10.1007/s11135-017-0574-8 29937585 PMC5993836

[cit0034] Schell, S. F., Luke, D. A., Schooley, M. W., Elliott, M. B., Herbers, S. H., Mueller, N. B., & Bunger, A. C. (2013). Public health program capacity for sustainability: A new framework. *Implementation Science*, 8(1), 1–9. 10.1186/1748-5908-8-15 23375082 PMC3599102

[cit0035] Shelton, R. C., & Lee, M. (2019). Sustaining evidence-based interventions and policies: Recent innovations and future directions in implementation science. *American Journal of Public Health*, 109(S2), S132–4. 10.2105/AJPH.2018.304913 30785794 PMC6383970

[cit0036] Taylor, A., Pilkington, R., Montgomerie, A., & Feist, H. (2016). The role of business size in assessing the uptake of health promoting workplace initiatives in Australia. *BMC Public Health*, 16(1), 353. 10.1186/s12889-016-3011-3 27097738 PMC4839116

[cit0037] Waterworth, P., Pescud, M., Chappell, S., Davies, C., Roche, D., Shilton, T., Ledger, M., Slevin, T. & Rosenberg, M. (2016). Culture, management and finances as key aspects for healthy workplace initiatives. *Health Promotion International*, 33(1), 162–172. 10.1093/heapro/daw068 27543456

[cit0038] Welsby, D., Nguyen, B., O’Hara, B. J., Innes-Hughes, C., Bauman, A., & Hardy, L. L. (2014). Process evaluation of an up-scaled community based child obesity treatment program: NSW Go4Fun®. *BMC Public Health*, 14(1), 140. 10.1186/1471-2458-14-140 24512080 PMC3923092

[cit0039] Wiggers, J., Wolfenden, L., Campbell, E., Gillham, K., Bell, C., Sutherland, R., Hardy, L. L., King, L., Grunseit, A., Milat, A. J., & Orr, N. (2013). *Good for kids, good for life 2006–2010: Evaluation report*. NSW Ministry of Health. 1741879035.

[cit0040] The World Bank. (2017, 6 9). *Total labor force*. World Bank. http://data.worldbank.org/indicator/SL.TLF.TOTL.IN?contextual=default

[cit0041] World Health Organization. (2017). *Noncommunicable diseases progress monitor.* Geneva: World Health Organization. (Contract No.: Licence: CC BY-NC-SA 3.0 IGO).

[cit0042] World Health Organization, & Burton, J. (2010). *WHO healthy workplace framework and model: Background and supporting literature and practices*. World Health Organization. 9241500247.

